# Outcomes of On- Versus Off-Pump Coronary Artery Bypass Grafting for Acute and Subacute Myocardial Infarction: A Nationwide Propensity Score-Matched Cohort Study

**DOI:** 10.3390/jcm15176707

**Published:** 2026-08-29

**Authors:** Hyo-Hyun Kim, Jiyoung Shin, Kang Ju Son, Kyung-Jong Yoo, Young-Nam Youn

**Affiliations:** 1 Department of Thoracic & Cardiovascular Surgery, National Health Insurance Service Ilsan Hospital, Goyang 10444, Republic of Korea; ysgs@naver.com (H.-H.K.);; 2 Research and Analysis Team, National Health Insurance Service Ilsan Hospital, Goyang 10444, Republic of Korea; 3 Division of Cardiovascular Surgery, Department of Thoracic and Cardiovascular Surgery, Soonchunhyang University Seoul Hospital, Soonchunhyang University College of Medicine, Seoul 04401, Republic of Korea; 4 Division of Cardiovascular Surgery, Severance Cardiovascular Hospital, Yonsei University College of Medicine, Yonsei University Health System, Seoul 03722, Republic of Korea

**Keywords:** acute myocardial infarction, coronary artery bypass grafting, off-pump CABG, propensity score matching, mortality, major adverse cardiovascular and cerebrovascular events

## Abstract

**Background/Objectives:** The comparative efficacy of off-pump and on-pump coronary bypass grafting (CABG) for patients with acute and subacute myocardial infarction (AMI) remains controversial. We compared the outcomes of on-pump versus off-pump coronary artery bypass grafting in Korean patients with acute and subacute myocardial infarction. **Methods:** This nationwide retrospective cohort study included 14,578 propensity score-matched patients undergoing on-pump or off-pump CABG between 2007 and 2022 using the Korean National Health Insurance Service database. The primary outcomes were recurrent myocardial infarction, repeat revascularization, stroke, all-cause mortality, and major adverse cardiovascular and cerebrovascular events. Chi-square, Kaplan–Meier, and log-rank tests and Cox regression analysis were used. **Results:** Compared with on-pump CABG, off-pump CABG was associated with significantly lower risks of major adverse cardiovascular and cerebrovascular events (MACCEs), all-cause mortality, and recurrent myocardial infarction in the cause-specific Cox analysis, whereas repeat revascularization and stroke rates were comparable between groups. However, the association with recurrent myocardial infarction was not statistically significant in the Fine–Gray competing-risk analysis. **Conclusions:** Off-pump CABG was associated with lower risks of all-cause mortality and MACCEs in patients with acute and subacute myocardial infarction. The association with recurrent myocardial infarction was less consistent across analytical approaches and should be interpreted cautiously.

## 1. Introduction

Acute myocardial infarction (AMI) remains one of the leading causes of cardiovascular mortality worldwide despite advances in coronary revascularization strategies. According to the National Center for Health Statistics, the incidence of acute myocardial infarction (AMI) per 1000 individuals rose from 1.36 in 2011 to 1.73 in 2015. Moreover, the cerebrovascular disease-associated mortality rate, including ischemic heart disease (IHD), is second only to cancer [1,2,3].

Recent advances in coronary artery disease treatment have improved surgical techniques and cardiopulmonary bypass (CPB) management for on-pump coronary artery bypass grafting (CABG). Meanwhile, off-pump CABG, which avoids CPB and its associated physiological effects, has been widely adopted as an alternative strategy [4,5,6]. However, the comparative outcomes of on-pump and off-pump CABG remain debated, particularly with respect to long-term clinical outcomes.

Randomized trials have yielded conflicting results. The ROOBY trial reported less favorable long-term outcomes and graft patency with off-pump CABG [7], whereas the CORONARY and GOPCABE trials found no clear long-term survival advantage of either strategy [8,9]. These findings suggest that the relative outcomes of off-pump and on-pump CABG may depend on patient selection and clinical setting.

Off-pump CABG remains relatively common in several Asian healthcare systems and high-volume centers, whereas on-pump CABG predominates in many Western settings. Given these differences in practice patterns and the limited nationwide evidence specifically addressing patients with acute or subacute myocardial infarction, we evaluated the long-term outcomes associated with on-pump versus off-pump CABG using a nationwide Korean cohort with propensity score matching.

## 2. Materials and Methods

### 2.1. Data Source

This retrospective study used customized data provided by the National Health Insurance Service (NHIS-2023-1-626) on patients who underwent on-pump or off-pump CABG (Supplementary Table S1). The NHIS is a mandatory universal health insurance system covering nearly the entire Korean population and contains comprehensive information on demographic characteristics, diagnoses, procedures, prescriptions, and mortality. Diagnoses were identified using the International Classification of Diseases, 10th Revision (ICD-10), and surgical and procedural information was obtained using NHIS procedure codes.

This study was approved by the Institutional Review Board of NHIS Ilsan Hospital (NHIMC-2023-07-006), which waived the requirement for informed consent owing to the anonymization of personal information. The study was registered at Research Registry (UIN: researchregistry10666, www.researchregistry.com) in accordance with the World Medical Association’s Declaration of Helsinki, 2013, and has been reported to be in line with the STROCSS criteria [10,11].

### 2.2. Study Population

Among 2,638,497 individuals diagnosed with IHD from 2005 to 2022, those who were diagnosed in 2005 and 2006 and underwent CABG or intervention during those years were excluded, resulting in a cohort of 18,835 individuals who underwent CABG within 1 year of qualifying MI diagnosis.

The nationwide IHD population served as the broader source population for cohort identification. Study population identification was based on predefined IHD diagnostic codes (I21, I22, I23, I25.0, and I25.1), as detailed in Supplementary Table S1. Within the study cohort, MI type was further classified as STEMI or NSTEMI, with STEMI identified using ICD-10 codes I21.0–I21.4 (Supplementary Table S3). Only patients who underwent CABG within one year of their qualifying diagnosis were included to ensure a clinically relevant and homogeneous cohort.

After applying the additional exclusion criteria, including a combination of CPB and non-CPB procedures, lack of sociodemographic data, and age < 30 years, 17,447 patients remained eligible for propensity score matching, including 9480 patients who underwent off-pump CABG and 7967 patients who underwent on-pump CABG.

**Figure 1:**
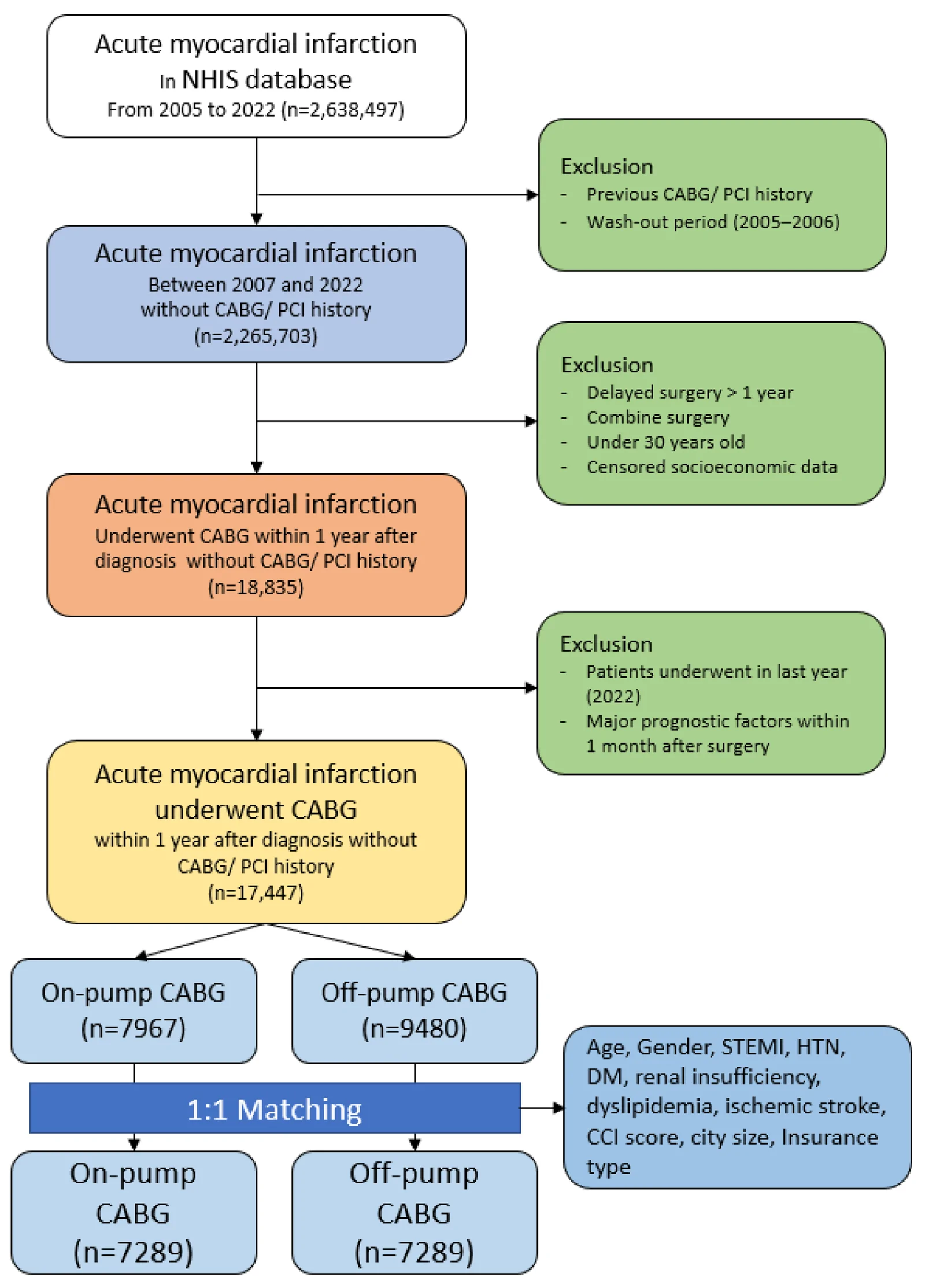
Study flow diagram showing patient selection. CABG, conventional coronary artery bypass surgery; CCI, Charlson comorbidity index; HTN, hypertension; NHIS, National Health Insurance Service; PCI, percutaneous coronary intervention; STEMI, ST-segment elevation myocardial infarction.

After 1:1 propensity score matching, the final matched cohort comprised 7289 patients who underwent on-pump CABG and 7289 patients who underwent off-pump CABG (Figure 1). Thus, 14,578 of the 17,447 eligible patients were retained, whereas 2869 patients (16.4%) were not retained in the propensity score-matched cohort.

### 2.3. Clinical Outcomes

The primary outcome was the time from enrollment to the occurrence of major adverse cardiovascular and cerebrovascular events (MACCEs), defined as a composite of recurrent myocardial infarction, repeat revascularization, stroke, and all-cause mortality. For the composite endpoint, the first occurrence of any component was considered a MACCE (Supplementary Table S2). Recurrent myocardial infarction was identified using the corresponding diagnostic codes for a new myocardial infarction event after the index CABG. To distinguish subsequent events from claims or procedures potentially related to the index CABG episode, recurrent myocardial infarction and repeat revascularization events occurring within 30 days after the index surgery were excluded. Accordingly, follow-up for these recurrent events began 30 days after CABG. Repeat revascularization was defined as PCI or repeat CABG occurring more than 30 days after the index CABG.

We recorded comorbidities, medications, and demographic characteristics potentially influencing prognostic variables (Supplementary Table S3): age, sex, city of residence, insurance quintile, diagnoses of hypertension, diabetes, renal insufficiency, dyslipidemia, and ischemic or hemorrhagic stroke within 2 years prior to CABG, and the presence of ST-segment elevation MI (STEMI)-related IHD. Overall severity was assessed using the Charlson comorbidity index, and medication prescriptions affecting prognosis were reviewed.

### 2.4. Statistical Analysis

Continuous variables were expressed as means with standard deviations, and categorical variables were expressed as frequencies and percentages. Differences between groups were compared using appropriate statistical tests according to the distribution and type of each variable.

PSM was performed to minimize potential confounding between patients undergoing off-pump and on-pump CABG. Propensity scores were estimated using logistic regression based on baseline covariates, including demographic characteristics, comorbidities, medication use, Charlson Comorbidity Index, residential area, and income level. Patients in the off-pump and on-pump CABG groups were matched in a 1:1 ratio without replacement using the SAS OneToManyMTCH macro, which employs a greedy 8-to-1 digit matching algorithm based on the propensity score.

Covariate balance was evaluated both before and after propensity score matching using standardized mean differences (SMDs), with an absolute SMD < 0.1 considered indicative of acceptable balance. Baseline characteristics of the unmatched cohort are presented in Supplementary Table S4, and those of the propensity score-matched cohort are presented in Table 1.

The distributions of propensity scores were visually examined before and after matching. Variance ratios were additionally calculated for continuous covariates before and after matching as an additional measure of covariate balance. For categorical variables, balance was assessed using SMDs because variables with multiple categories cannot be summarized by a single conventional variance ratio.

Absolute SMDs for individual covariates before and after matching were graphically summarized using a Love plot to provide a more direct visualization of changes in covariate balance after matching. No multiple imputation was performed. Observations with missing values were excluded from the corresponding analyses, and analyses were performed using available complete cases.

Kaplan–Meier survival curves were generated to estimate cumulative event-free survival, and differences between groups were assessed using the log-rank test. Cox proportional hazards regression models were used to estimate hazard ratios (HRs) and 95% confidence intervals (CIs) for clinical outcomes.

Because death may preclude the occurrence of nonfatal cardiovascular outcomes, competing-risk analyses were additionally performed using Fine–Gray subdistribution hazard models, with death treated as a competing event where appropriate. Subdistribution hazard ratios and corresponding 95% CIs were estimated.

Statistical analyses were performed using SAS software (version 9.4; SAS Institute Inc., Cary, NC, USA). All statistical tests were two-sided, and a *p* value < 0.05 was considered statistically significant.

## 3. Results

### 3.1. Baseline Characteristics

The matched cohort had a mean age of 64.9 ± 9.9 years, and 72.2% of the patients were male. Baseline characteristics of the unmatched cohort are presented in Supplementary Table S4, and those of the propensity score-matched cohort are presented in Table 1. Propensity score matching improved the overall balance of measured baseline covariates between the two groups, although residual imbalances remained for some variables.

### 3.2. Propensity Score Matching Performance

**Figure 2:**
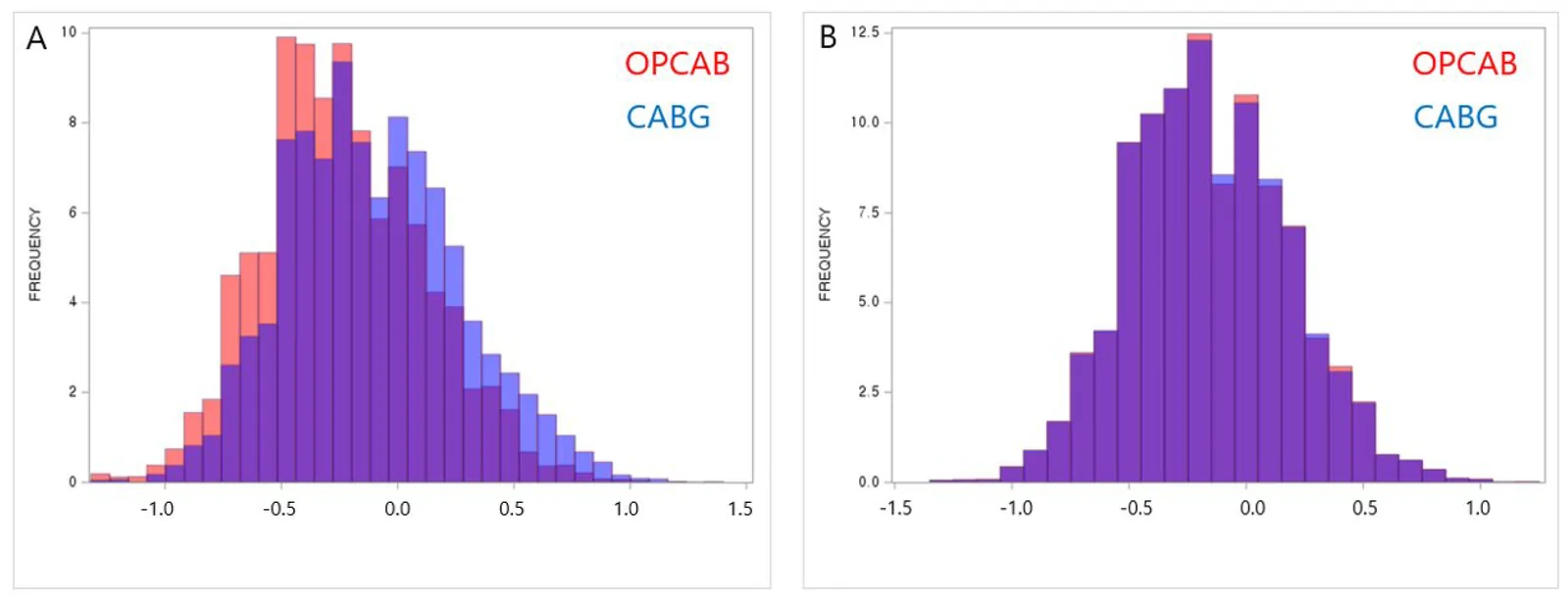
Distribution of propensity scores before and after propensity score matching. Histogram plots showing the distribution of propensity scores before (**A**) and after (**B**) propensity score matching. PSM, propensity score matching.

The distribution of propensity scores before and after matching is shown in Figure 2. Before matching, the on-pump and off-pump CABG groups demonstrated different propensity score distributions. Following propensity score matching, substantial overlap was achieved, indicating improved covariate balance between the treatment groups (Figure 2A,B).

**Figure 3:**
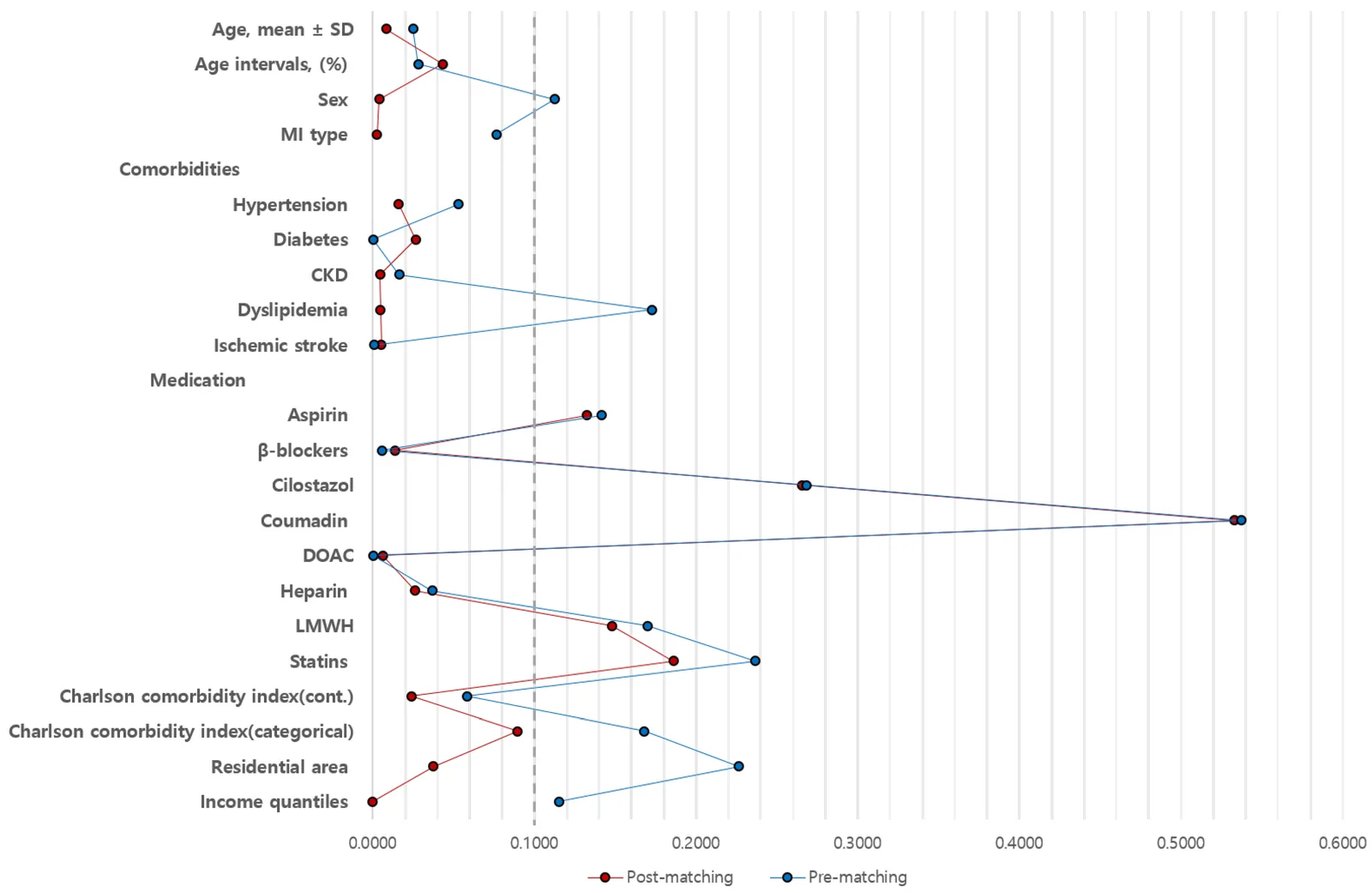
Love plot of covariate balance before and after propensity score matching. Absolute standardized mean differences (SMDs) for baseline covariates are shown before and after matching. The vertical dashed line at an absolute SMD of 0.1 indicates the prespecified threshold for acceptable covariate balance.

The Love plot further demonstrated an overall improvement in covariate balance after matching, although residual imbalances remained for some covariates (Figure 3).

### 3.3. Long-Term Clinical Outcomes

**Figure 4:**
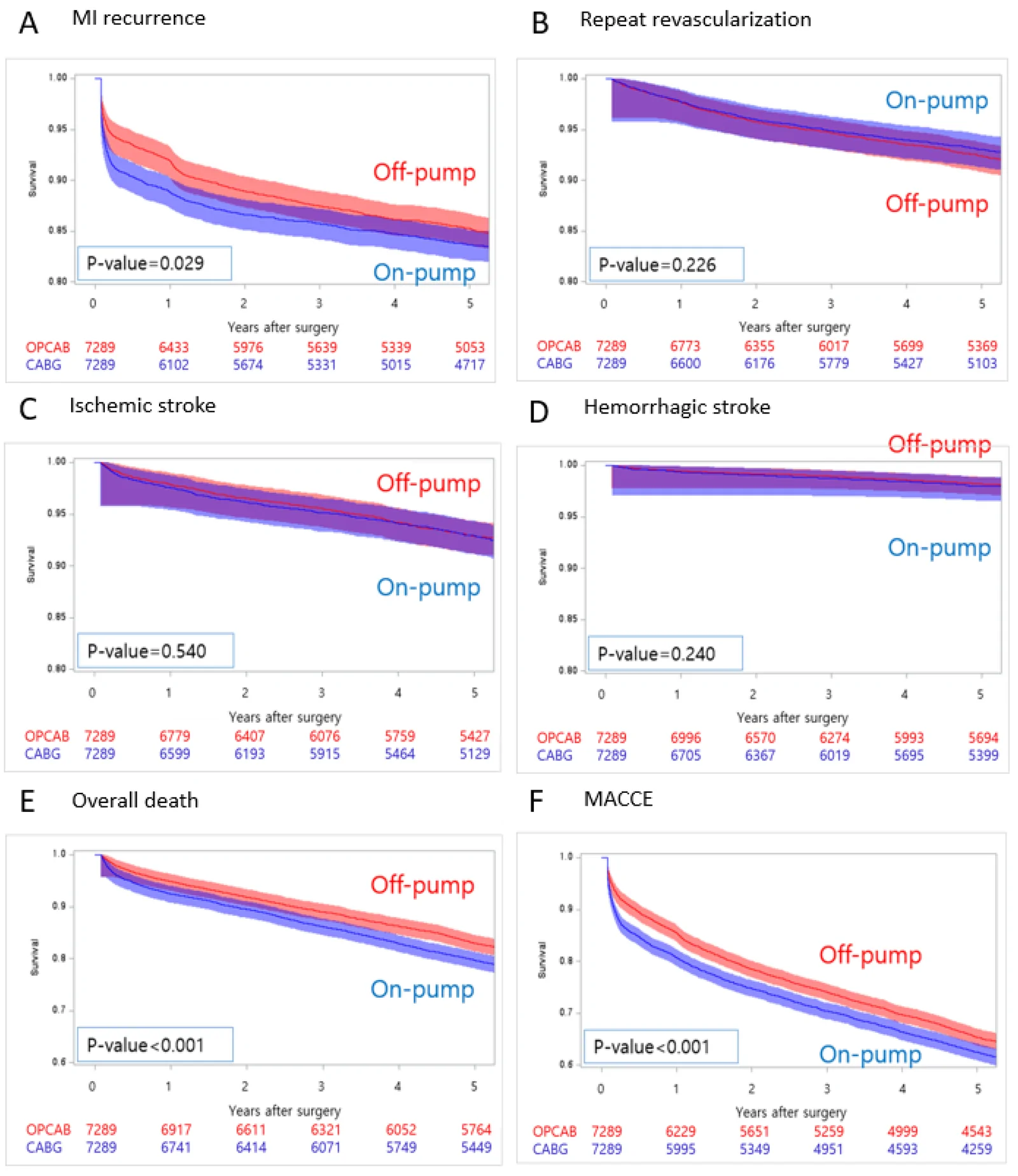
Kaplan–Meier curves for long-term clinical outcomes after propensity score matching. Kaplan–Meier curves comparing (**A**) recurrent myocardial infarction, (**B**) repeat revascularization, (**C**) ischemic stroke, (**D**) hemorrhagic stroke, (**E**) all-cause mortality, and (**F**) major adverse cardiovascular and cerebrovascular events (MACCEs) between the off-pump and on-pump CABG groups after propensity score matching. MACCE, major adverse cardiac and cerebrovascular event; PS, propensity score.

The median follow-up duration was 3.1 years (IQR, 1.6–4.5 years), corresponding to a total of 45,192 person-years of observation. Kaplan–Meier analyses demonstrated significant differences in several long-term outcomes between the two treatment groups (Figure 4). Kaplan–Meier analysis showed a lower cumulative incidence of recurrent myocardial infarction in the off-pump CABG group than in the on-pump CABG group (log-rank *p* = 0.029) (Figure 4A), although this finding was not consistent in the competing-risk analysis described below. Overall survival was significantly higher in the off-pump CABG group (log-rank *p* < 0.001) (Figure 4E). The cumulative incidence of MACCEs was also significantly lower in the off-pump CABG group throughout follow-up (log-rank *p* < 0.001) (Figure 4F).

In contrast, no significant differences were observed between the two groups regarding repeat revascularization (log-rank *p* = 0.226), ischemic stroke (log-rank *p* = 0.540), or hemorrhagic stroke (log-rank *p* = 0.240) (Figure 4B–D).

### 3.4. Survival and Competing-Risk Analyses

The results of the cause-specific Cox proportional hazards model and the Fine–Gray competing-risk model are summarized in Table 2. Absolute event counts and rates for each treatment group are also presented in Table 2 and Supplementary Table S5. All-cause mortality occurred in 2541 patients (34.86%) in the off-pump group and 2860 patients (39.24%) in the on-pump group. Off-pump CABG was associated with a lower risk of all-cause mortality (HR, 0.865; 95% CI, 0.820–0.912; *p* < 0.001).

Recurrent myocardial infarction occurred in 1314 patients (18.03%) in the off-pump group and 1378 patients (18.91%) in the on-pump group. In the cause-specific Cox model, off-pump CABG was associated with a lower risk of recurrent myocardial infarction (HR, 0.919; 95% CI, 0.852–0.991; *p* = 0.029), although this association was attenuated and was no longer statistically significant in the Fine–Gray competing-risk model (HR, 0.936; 95% CI, 0.868–1.010; *p* = 0.087).

Repeat revascularization occurred in 798 patients (10.95%) in the off-pump group and 731 patients (10.03%) in the on-pump group; ischemic stroke occurred in 815 (11.18%) and 767 (10.52%), respectively; and hemorrhagic stroke occurred in 182 (2.50%) and 199 (2.73%), respectively. No significant differences were observed for repeat revascularization, ischemic stroke, or hemorrhagic stroke in either model.

MACCEs occurred in 3848 patients (52.79%) in the off-pump group and 4074 patients (55.89%) in the on-pump group. Off-pump CABG was associated with a lower risk of MACCEs in both the cause-specific Cox model (HR, 0.905; 95% CI, 0.866–0.945; *p* < 0.001) and the Fine–Gray model (HR, 0.890; 95% CI, 0.826–0.958; *p* = 0.005).

### 3.5. Temporal Trends in AMI and CABG

Temporal trends in acute myocardial infarction diagnoses and CABG utilization are presented in Supplementary Figure S1. The annual number of patients diagnosed with acute myocardial infarction gradually increased between 2011 and 2019, followed by a decline during the COVID-19 pandemic. Similarly, the annual number of CABG procedures decreased after 2017 (Supplementary Figure S1).

## 4. Discussion

The principal finding of this nationwide propensity score-matched cohort study is that off-pump CABG was associated with significantly lower risks of all-cause mortality and MACCEs than on-pump CABG in patients undergoing surgical revascularization for acute and subacute myocardial infarction. Notably, no significant differences were observed in repeat revascularization or ischemic and hemorrhagic stroke. For recurrent myocardial infarction, a significantly lower risk was observed with off-pump CABG in the cause-specific Cox model; however, this association was attenuated and was no longer statistically significant in the Fine–Gray competing-risk analysis. Therefore, the association with recurrent myocardial infarction should be interpreted cautiously. The associations with all-cause mortality and MACCEs were consistent across the analytical approaches.

The comparative effectiveness of off-pump and on-pump CABG has remained controversial for more than two decades. The ROOBY trial reported inferior long-term graft patency and increased adverse cardiac events after off-pump CABG, whereas the CORONARY and GOPCABE trials demonstrated comparable long-term clinical outcomes between the two techniques [7,8,9,12,13]. Furthermore, observational studies and meta-analyses have produced inconsistent results, with some reporting improved perioperative outcomes but reduced completeness of revascularization after off-pump CABG, whereas others found no clinically meaningful differences in long-term survival [14,15,16,17,18].

In contrast to several earlier studies, our nationwide cohort demonstrated lower long-term mortality and MACCEs after off-pump CABG without an increase in repeat revascularization. However, off-pump and on-pump CABG are not necessarily interchangeable strategies across all patients in routine clinical practice. Surgical strategy may be influenced by individual patient characteristics, coronary anatomy, lesion complexity, and the extent of revascularization required, with on-pump CABG potentially being selected for patients requiring more extensive or anatomically complex revascularization. Because these factors were not available in the NHIS database, the observed differences in outcomes may partly reflect treatment selection and confounding by indication rather than the surgical strategy itself. Therefore, these findings should be interpreted as associations rather than evidence of the superiority of one surgical strategy over the other.

The observed association with lower mortality in the present cohort contrasts with randomized evidence, which has not consistently demonstrated a long-term survival advantage of off-pump CABG. This discrepancy may reflect differences in patient populations, treatment selection, and patterns of surgical practice between randomized trials and real-world cohorts. One possible explanation is the relatively high level of institutional and surgeon experience with off-pump CABG in South Korea; however, surgeon-level and hospital-level procedural volume were unavailable in the NHIS dataset, and this hypothesis could not be directly tested. Residual confounding and treatment-selection bias therefore remain important alternative explanations for the observed mortality association.

The magnitude of the observed mortality association was modest (HR, 0.865), corresponding to an approximately 13.5% lower relative hazard rather than a large survival difference. In a nationwide cohort with prolonged follow-up, such a difference may nevertheless be clinically meaningful, particularly among patients at relatively high baseline cardiovascular risk. However, given the observational nature of the study and the potential for residual confounding and treatment-selection bias, the observed mortality association should be interpreted cautiously and should not be considered evidence of a causal survival benefit of off-pump CABG.

Several potential mechanisms may nevertheless contribute to the observed associations. Cardiopulmonary bypass induces systemic inflammatory activation, hemodilution, coagulation disturbances, and embolic phenomena, all of which may contribute to perioperative myocardial and neurological injury [19,20]. Avoidance of cardiopulmonary bypass has been proposed as a potential mechanism for reducing perioperative myocardial injury and preserving postoperative cardiac function.

Another important consideration is the unique surgical environment in South Korea. Unlike many Western countries, off-pump CABG has been widely adopted since the 1990s and remains the preferred revascularization strategy in many high-volume centers. Consequently, Korean cardiac surgeons have accumulated extensive experience with off-pump techniques, and previous Korean studies have reported favorable outcomes with this approach [16,21]. In addition, the concentration of cardiac surgery in experienced tertiary referral hospitals may further contribute to favorable surgical outcomes [22]. Therefore, the present findings should be interpreted within the context of a healthcare system in which surgeon experience and institutional volume strongly support the routine performance of off-pump CABG. Accordingly, the observed associations may not be directly generalizable to healthcare systems with limited institutional or surgeon experience in off-pump CABG.

From a clinical perspective, the incidence of acute myocardial infarction continues to increase in the aging Korean population, emphasizing the importance of selecting an optimal surgical strategy for high-risk patients [23]. The present findings suggest that off-pump CABG may be associated with favorable long-term outcomes, particularly in healthcare systems where this technique is routinely practiced.

Several limitations should be acknowledged. First, this was a retrospective observational study, and residual confounding cannot be completely excluded despite rigorous propensity score matching and competing-risk analyses. Second, the NHIS database does not contain detailed anatomical or operative information, including coronary anatomy, left ventricular function, graft patency, number of grafts, graft configuration, conduit selection, completeness of revascularization, or surgeon experience. In addition, information on lesion complexity, cardiogenic shock, Killip class, infarct size, the exact timing of CABG after MI, institutional CABG volume, surgical urgency, and intraoperative conversion from off-pump to on-pump CABG was unavailable. Consequently, we were unable to stratify patients according to the timing of CABG after MI or perform sensitivity analyses restricted to patients undergoing CABG during the index hospitalization or within a prespecified acute period. The inclusion of patients undergoing CABG within 1 year of the qualifying diagnosis may therefore have introduced clinical heterogeneity and selection related to the timing of surgery. Importantly, these unmeasured clinical, anatomical, and procedural factors may have influenced not only subsequent clinical outcomes but also the selection of off-pump versus on-pump CABG. Therefore, residual confounding by indication and selection bias may remain despite propensity score matching, and the observed associations should not be interpreted as establishing a causal benefit of off-pump CABG. Third, institutional preferences and referral patterns unique to South Korea may limit the generalizability of these findings to other healthcare systems. Finally, although important demographic and socioeconomic variables were adjusted for, unmeasured confounders may still have influenced the observed associations.

## 5. Conclusions

In this nationwide propensity score-matched cohort study, off-pump coronary artery bypass grafting was associated with lower risks of all-cause mortality and major adverse cardiac and cerebrovascular events than on-pump coronary artery bypass grafting in patients with acute and subacute myocardial infarction. The association with recurrent myocardial infarction was significant in the cause-specific Cox analysis but not in the Fine–Gray competing-risk analysis and should therefore be interpreted cautiously. These findings suggest that off-pump CABG may be associated with favorable long-term clinical outcomes without increasing the risks of repeat revascularization or stroke when performed in experienced centers. However, these findings should be interpreted within the context of the extensive off-pump CABG experience in South Korea and may not be directly generalizable to healthcare systems where off-pump CABG is performed less frequently. Prospective studies are warranted to confirm these findings and further define the optimal surgical strategy for this high-risk population.

## Figures and Tables

**Table 1:** Baseline characteristics of the propensity score-matched study population.

Characteristics	Overall *n* = 14,578	Off-Pump CABG *n* = 7289	On-Pump CABG *n* = 7289	Standard Difference
Age, mean ± SD	64.93 ± 9.94	64.97 ± 9.86	64.89 ± 10.02	−0.008
Age intervals, (%)				0.043
30–39 years	139 (0.95)	66 (0.91)	73 (1.00)	
40–49 years	940 (6.45)	459 (6.30)	481 (6.60)	
50–59 years	3107 (21.31)	1565 (21.47)	1542 (21.16)	
60–69 years	5124 (35.15)	2558 (35.09)	2566 (35.20)	
70–79 years	4572 (31.36)	2306 (31.64)	2266 (31.09)	
≥80 years	696 (4.77)	335 (4.60)	361 (4.95)	
Sex				−0.004
Males	10,523 (72.18)	5268 (72.27)	5255 (72.09)	
Females	4055 (27.82)	2021 (27.73)	2034 (27.91)	
MI type				−0.003
STEMI	608 (4.17)	306 (4.2)	302 (4.14)	
NSTEMI	13,970 (95.83)	6983 (95.8)	6987 (95.86)	
Comorbidities				
Hypertension	12,830 (88.01)	6434 (88.27)	6396 (87.75)	−0.016
Diabetes	9568 (65.63)	4830 (66.26)	4738 (65.00)	−0.027
CKD	1175 (8.06)	592 (8.12)	583 (8.00)	−0.005
Dyslipidemia	11,658 (79.97)	5836 (80.07)	5822 (79.87)	−0.005
Ischemic stroke	3366 (23.09)	1675 (22.98)	1691 (23.20)	0.005
Medication				
Aspirin	14,507 (99.51)	7287 (99.97)	7220 (99.05)	−0.132
β-blockers	10,369 (71.13)	5207 (71.44)	5162 (70.82)	−0.014
Cilostazol	13,891 (95.29)	7149 (98.08)	6742 (92.50)	−0.266
Coumadin	2850 (19.55)	681 (9.34)	2169 (29.76)	0.533
DOAC	181 (1.24)	93 (1.28)	88 (1.21)	−0.006
Heparin	40 (0.27)	15 (0.21)	25 (0.34)	0.026
LMWH	5776 (39.62)	2625 (36.01)	3151 (43.23)	0.148
Statins	13,470 (92.40)	6914 (94.86)	6556 (89.94)	−0.186
Charlson comorbidity index				
Mean ± SD	4.55 ± 2.50	4.58 ± 2.51	4.52 ± 2.49	−0.024
0	219 (1.50)	102 (1.4)	117 (1.61)	0.090
1–2	3010 (20.65)	1470 (20.17)	1540 (21.13)	
3–4	4636 (31.80)	2324 (31.88)	2312 (31.72)	
≥5	6713 (46.05)	3393 (46.55)	3320 (45.55)	
Residential area				0.037
Capital	3272 (22.44)	1623 (22.27)	1649 (22.62)	
Metropolitan	3066 (21.03)	1524 (20.91)	1542 (21.16)	
City	6728 (46.15)	3386 (46.45)	3342 (45.85)	
Rural	1512 (10.37)	756 (10.37)	756 (10.37)	
Income quantiles				0.001
Medical aid (lowest)	885 (6.07)	443 (6.08)	442 (6.06)	
First quartile	2629 (18.03)	1315 (18.04)	1314 (18.03)	
Second quartile	2353 (16.14)	1171 (16.07)	1182 (16.22)	
Third quartile	3341 (22.92)	1659 (22.76)	1682 (23.08)	
Fourth quartile	5370 (36.84)	2701 (37.06)	2669 (36.62)	

Data are presented as mean ± standard deviation or *n* (%), as appropriate. Standardized mean differences (SMDs) were used to assess covariate balance between the off-pump and on-pump CABG groups, with an absolute SMD < 0.10 considered indicative of acceptable balance. CABG, coronary artery bypass grafting; CKD, chronic kidney disease; DOAC, direct oral anticoagulant; LMWH, low-molecular-weight heparin; SMD, standardized mean difference.

**Table 2:** Absolute event rates and comparison of cause-specific Cox and Fine–Gray competing-risk models for long-term clinical outcomes.

Outcome	Off-Pump CABG, *n* (%)	On-Pump CABG, *n* (%)	Cause-Specific Cox Model HR (95% CI)	*p* Value	Fine and Gray Model HR (95% CI)	*p* Value
Overall death	2541 (34.86)	2860 (39.24)	0.865 (0.820–0.912)	*p* < 0.001	-	
Myocardial infarction recurrence	1314 (18.03)	1378 (18.91)	0.919 (0.852–0.991)	*p* = 0.029	0.936 (0.868–1.010)	*p* = 0.087
Repeat revascularization	798 (10.95)	731 (10.03)	1.064 (0.962–1.176)	*p* = 0.226	1.102 (0.997–1.218)	*p* = 0.058
Ischemic stroke	815 (11.18)	767 (10.52)	1.031 (0.934–1.138)	*p* = 0.540	1.068 (0.968–1.178)	*p* = 0.191
Hemorrhagic stroke	182 (2.50)	199 (2.73)	0.886 (0.725–1.084)	*p* = 0.240	0.915 (0.749–1.119)	*p* = 0.388
MACCE	3848 (52.79)	4074 (55.89)	0.905 (0.866–0.945)	*p* < 0.001	0.890 (0.826–0.958)	*p* = 0.005

Data are presented as *n* (%) or hazard ratio (95% confidence interval). Hazard ratios represent off-pump CABG relative to on-pump CABG. For nonfatal outcomes, death before the occurrence of the outcome of interest was treated as a competing event in the Fine–Gray model. MACCEs, major adverse cardiac and cerebrovascular events; CABG, coronary artery bypass grafting.

## Data Availability

The source data underlying all figures are publicly available in Mendeley Data (https://doi.org/10.17632/khcjtcskdw.1). Additional de-identified data supporting the findings of this study are available from the corresponding author upon reasonable request, subject to approval by the Korean National Health Insurance Service and applicable institutional and ethical regulations. Individual-level clinical data cannot be publicly shared because informed consent for unrestricted data sharing was not obtained and access is governed by data protection regulations.

## Supplementary Materials

The following supporting information can be downloaded at: https://www.mdpi.com/article/10.3390/jcm15176707/s1, Figure S1. Annual trends in first-time acute myocardial infarction (AMI) or ST-elevation myocardial infarction (STEMI) (A) and the number of coronary artery bypass grafting (CABG) procedures performed in South Korea since 2017 (B); Table S1. Criteria codes used for study population identification; Table S2. Definitions of clinical endpoints; Table S3. Definitions of comorbidities and medications; Table S4. Baseline characteristics of the pre-matching study population; Table S5. Absolute frequencies of long-term clinical outcomes after off-pump versus on-pump CABG in the propensity score-matched cohort.
