# Supplementary material for: Outcomes of On- Versus Off-Pump Coronary Artery Bypass Grafting for Acute and Subacute Myocardial Infarction: A Nationwide Propensity Score-Matched Cohort Study
Source: J Clin Med. 2026 Aug 29;15(17):6707. doi: 10.3390/jcm15176707 (PMC13566332; doi:10.3390/jcm15176707)
Supplement: Supplementary file 1 [file jcm-15-06707-s001.zip › jcm-4492947-supplementary.pdf]

Supplementary Figure S1. Annual trends in first-time acute myocardial infarction (AMI) or ST-elevation myocardial infarction (STEMI) (A) and the number of coronary artery bypass grafting (CABG) procedures performed in South Korea since 2017 (B).

A

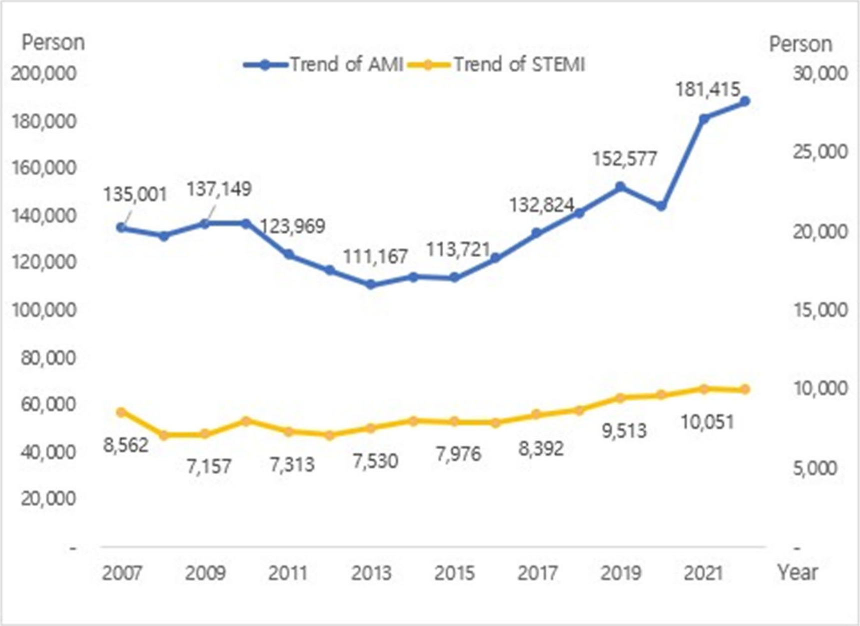

B

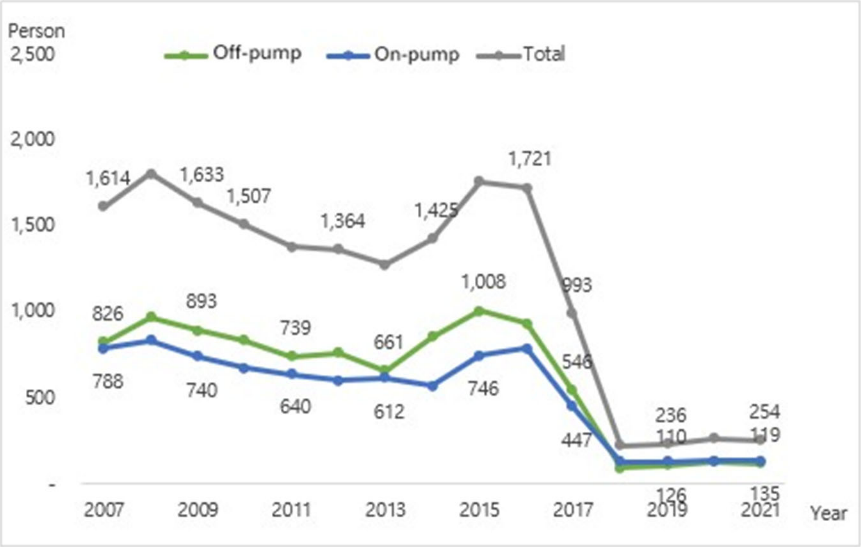

Supplementary Table S1. Criteria codes used for study population identification.

Variables	Codes
Diagnostic code	Ischemic heart disease
	I21, I22, I23, I250, I251
Surgery code	On-pump coronary artery bypass (CABG)
	O1641, O1642, O1647
	Off-pump coronary artery bypass (OPCAB)
	OA641, OA642, OA647

Supplementary Table S2. Definitions of clinical endpoints.

Variables	Codes
Repeat myocardial infarction (MI)	I21, I22, I23, I250, I251
Repeat revascularization	
Re-coronary artery bypass surgery	A1641, O1642, O1647, OA641, OA642, OA647
Repeat coronary reintervention	M6551, M6552, M6561, M6562, M6563, M6564, M6571, M6572, M6633, M6634
Ischemic stroke	I63, I65, I66, I672, I673, I675, I676, I693
Hemorrhagic stroke	I60, I61, I620, I670, I671, I677, I690, I691, I692

Supplementary Table S3. Definitions of comorbidities and medications.

Variables	Codes
Hypertension	I10, I11, I12, I13, I15
Diabetes mellitus	E10, E11, E12, E13, E14
Renal failure	N18
Dyslipidemia	E780, E781, E782, E783, E784, E785
Ischemic stroke	I63, I65, I66, I672, I673, I675, I676, I693
Hemorrhagic stroke	I60, I61, I620, I670, I671, I677, I690, I691, I692
STEMI	I210, I211, I212, I213, I214
Preoperative medications	Aspirin, b-blocker, cilostazol, coumadin, DOAC, Heparin, LWMH, Statins
Socioeconomic character	Year, sex, city, insurance information

Supplementary Table S4. Baseline characteristics of the pre-matching study population.

Characteristics	Overall<br>N=17,447	OPCAB<br>N=9,480	CABG<br>N=7,967	Standard<br>difference
Age, mean ± SD	64.75±10.04	64.63±10.02	64.88±10.07	0.0250
Age intervals, (%)				0.0282
30-39 years	180 (1.03)	96 (1.01)	84 (1.05)	
40-49 years	1,196 (6.86)	664 (6.97)	535 (6.72)	
50-59 years	3,769 (21.6)	2,082 (21.96)	1687 (21.17)	
60-69 years	6,101 (34.97)	3,335 (35.18)	2766 (34.72)	
70-79 years	5,352 (30.68)	2,856 (30.13)	2496 (31.33)	
≥80 years	849 (4.87)	450 (4.75)	399 (5.01)	
Sex				-0.1124
Men	12,758 (73.12)	7,148 (75.4)	5,610 (70.42)	
Women	4,689 (26.88)	2,332 (24.6)	2,357 (29.58)	
MI type				0.0764
STEMI	746 (4.28)	338 (3.57)	408 (5.12)	
NSTEMI	16,701 (95.71)	9,142 (96.43)	7,559 (94.88)	
Comorbidities				
Hypertension	15,270 (87.52)	8,373 (88.32)	6,897 (86.57)	-0.0529
Diabetes	11,238 (64.41)	6,107 (64.42)	5,131 (64.4)	-0.0003
CKD	1,415 (8.11)	788 (8.31)	627 (7.87)	-0.0162
Dyslipidemia	13,955 (79.99)	7,882 (83.14)	6,073 (76.23)	-0.1725
Ischemic stroke	4,050 (23.21)	2,202 (23.23)	1,848 (23.2)	-0.0008
Medication				
Aspirin	17,358 (99.49)	9,477 (99.97)	7,881 (98.92)	-0.1413
β-blockers	12,322 (70.68)	6,712 (70.8)	5,620 (70.54)	-0.0057

Cilostazol	16,659 (95.48)	9,297 (98.07)	7,362 (92.41)	-0.2683
Coumadin	3287 (18.84)	892 (9.41)	2,395 (30.06)	0.5373
DOAC	206 (1.18)	112 (1.180	94 (1.18)	-0.0001
Heparin	51 (0.29)	19 (0.2)	32 (0.4)	0.0367
LMWH	6,718 (38.51)	3,293 (34.74)	3,425 (42.99)	0.1699
Statins	16,093 (92.24)	9,021 (95.16)	7,072 (88.77)	-0.2367
Charlson comorbidity index				
mean ± SD	4.45±2.52	4.38±2.56	4.53±2.46	0.0582
0	360 (2.06)	243 (2.56)	117 (1.47)	0.1681
1-2	3,828 (21.94)	2,203 (23.24)	1,625 (20.4)	
3-4	5,511 (31.59)	2,925 (30.85)	2,586 (32.46)	
≥5	7,748 (44.41)	4,109 (43.34)	3,639 (45.68)	
Residential area				0.2266
Capital	4,347 (24.92)	2,685 (28.32)	1,662 (20.86)	
Metropolitan	3,572 (20.47)	1,577 (16.64)	1,995 (25.04)	
City	7,794 (44.67)	4,308 (45.44)	3,486 (43.76)	
Rural	1,734 (9.94)	910 (9.6)	824 (10.34)	
Income quantiles				0.1151
Medical aid (lowest)	1,033 (5.92)	517 (5.45)	516 (6.48)	
first quartile	3,079 (17.65)	1,603 (16.91)	1,476 (18.53)	
Second quartile	2,752 (15.77)	1,408 (14.85)	1,344 (16.87)	
Third quartile	3,989 (22.86)	2,161 (22.80)	1,828 (22.94)	
Fourth quartile	6,594 (37.79)	3,791 (39.99)	2,803 (35.18)	

Supplementary Table S5. Absolute frequencies of long-term clinical outcomes after off-pump versus on-pump CABG in the propensity score-matched cohort

**Characteristics**	**Overall N=14,578**	**Off-pump N=7,289**	**On-pump N=7,289**	**P-value**
MACCEs	7,922 (54.34)	3,848 (52.79)	4,074 (55.89)	0.0002
Overall death	5,401 (37.05)	2,541 (34.86)	2,860 (39.24)	<0.0001
MI recurrence	2,692 (18.47)	1,314 (18.03)	1,378 (18.91)	0.1719
Repeat revascularization	1,529 (10.49)	798 (10.95)	731 (10.03)	0.0701
Ischemic stroke	1,582 (10.85)	815 (11.18)	767 (10.52)	0.2012
Hemorrhagic stroke	381 (2.61)	182 (2.50)	199 (2.73)	0.3775
